# Primary Bilateral Macronodular Adrenocortical Disease With Concomitant Cushing Syndrome and Primary Aldosteronism Harboring Distinct ARMC5 Mutations in Individual Nodules

**DOI:** 10.7759/cureus.102743

**Published:** 2026-01-31

**Authors:** Sohei Kitazawa, Riko Kitazawa

**Affiliations:** 1 Molecular Pathology, Ehime University Graduate School of Medicine, Toon, JPN; 2 Diagnostic Pathology, Ehime University Hospital, Toon, JPN

**Keywords:** adrenal glands, aldosteronism, armc5, cushing syndrome, secondary hypertension

## Abstract

Primary bilateral macronodular adrenocortical disease is an uncommon adrenal disorder characterized by bilateral adrenal enlargement with multiple nodules and autonomous steroid hormone production. Although genetic alterations involving ARMC5 have been implicated in its development, individual nodules within the same adrenal gland often show considerable variability in size, morphology, and hormonal activity, and detailed genetic evaluation of each nodule has rarely been performed. We report the case of a 58-year-old man with a long-standing history of hypertension who was found to have bilateral adrenal nodules on imaging studies. Endocrinological evaluation revealed primary aldosteronism with lateralized aldosterone secretion from the left adrenal gland, along with autonomous cortisol secretion from both adrenal glands. The patient underwent laparoscopic left adrenalectomy. Gross and histopathological examination demonstrated multiple macronodular lesions without evidence of malignancy. Genetic analyses conducted separately on individual nodules identified different ARMC5 mutations in each nodule examined, indicating intraglandular genetic heterogeneity. This case illustrates that distinct adrenal nodules within primary bilateral macronodular adrenal disease can arise from genetically independent clonal events. The concomitant presence of cortisol and aldosterone overproduction further underscores the functional diversity of this condition. Careful histopathological sampling combined with nodule-specific molecular analysis may provide important insights into the pathogenesis and biological heterogeneity of this disease.

## Introduction

Primary bilateral macronodular adrenocortical disease (PBMAD), formerly referred to as primary bilateral macronodular adrenocortical hyperplasia, is a rare cause of adrenocorticotropic hormone (ACTH)-independent adrenal hormone excess [1-3]. It is characterized by bilateral adrenal enlargement with multiple benign macronodules measuring ≥1 cm and heterogeneous, autonomous steroid secretion, most commonly resulting in cortisol overproduction [1,2,4-6]. Reflecting advances in the understanding of its pathogenesis, this condition has recently been reclassified from a subtype of adrenal hyperplasia to a distinct disease entity in the updated World Health Organization classification, highlighting its genetic and biological heterogeneity [2].

Among the genetic alterations implicated in PBMAD, inactivating mutations of armadillo repeat-containing 5 (ARMC5), a tumor suppressor gene, have been established as a major contributor to disease development [7-10]. Current evidence supports a two-hit mechanism, in which a germline ARMC5 mutation is followed by somatic second-hit events within adrenal tissue, leading to bilateral and multifocal adrenal hyperplasia [11]. Despite this genetic framework, PBMAD shows considerable heterogeneity at the morphological and functional levels [12], with individual nodules often differing in size, histological appearance, and steroidogenic activity [13].

However, genetic analyses performed separately on individual hyperplastic nodules obtained from surgical specimens remain scarce, and the clonal relationships among nodules within the same patient are not fully understood. Given the insidious clinical course and variable hormonal presentation of PBMAD, accumulation of well-documented clinicopathological cases remains important.

In the present report, we describe a patient with bilateral multinodular adrenal disease who underwent surgical resection, allowing detailed pathological examination and separate genetic analysis of multiple hyperplastic nodules.

## Case presentation

Case history

A 58-year-old man with marked obesity (height, 172 cm; body weight, approximately 104 kg; body mass index, 35 kg/m²) was referred to our institution for evaluation of bilateral adrenal nodules in March 2024. He had been diagnosed with hypertension at 38 years of age during a routine health examination and had since been treated with antihypertensive medication. Around the age of 50 years, he experienced an unintentional weight loss of approximately 3 kg, which prompted evaluation at a local clinic. Laboratory testing revealed hyperglycemia, and he was subsequently diagnosed with diabetes mellitus and started on oral hypoglycemic therapy. One year prior to referral, computed tomography revealed bilateral adrenal nodules measuring approximately 1.0 cm in diameter. Endocrinological evaluation demonstrated an elevated plasma aldosterone concentration, and a captopril challenge test yielded a positive result, raising suspicion of primary aldosteronism. The patient was therefore referred to our hospital for further assessment.

**Figure 1:**
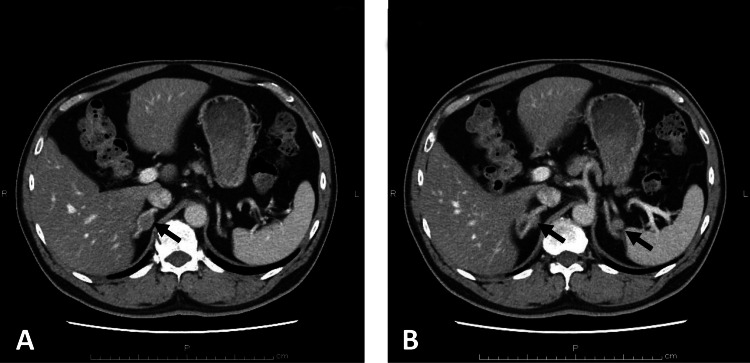
Contrast-enhanced computed tomography at initial presentation demonstrating The figure shows bilateral adrenal nodules (A, 19 mm; B, 12 mm, arrows) with relatively well-defined margins and slightly reduced enhancement compared with the surrounding adrenal tissue.

Contrast-enhanced computed tomography performed at the time of initial presentation (Figure 1) revealed nodular lesions in both adrenal glands. The nodules measured 19 mm in maximal diameter on the right (A) and 12 mm on the left (B). Both lesions were relatively well circumscribed and showed slightly lower contrast enhancement than the surrounding adrenal parenchyma. No focal hepatic lesions were identified. An accessory spleen was present. Fine calcifications were observed in the pancreatic uncinate process and tail. There was no significant enlargement of para-aortic lymph nodes.

Laboratory data obtained at the time of the patient’s initial presentation are summarized in Table 1. 

**Table 1:** Laboratory findings at initial presentation Laboratory data obtained at the time of the patient’s initial presentation are summarized. Values are shown with the corresponding units and reference ranges. H indicates values higher than the reference range, and L indicates values lower than the reference range. ARC, active renin concentration; PAC, plasma aldosterone concentration; eGFR, estimated glomerular filtration rate; HbA1c, hemoglobin A1c (NGSP).

Test Item	Result	H/L	Unit	Reference Range
Total protein	6.6	-	g/dL	6.6–8.2
Albumin	4	-	g/dL	3.9–4.9
Globulin	2.6	-	g/dL	2.0–3.9
A/G ratio	1.5	-	-	1.0–2.0
Total bilirubin	0.6	-	mg/dL	0.1–1.1
Direct bilirubin	0.1	-	mg/dL	0.0–0.6
Indirect bilirubin	0.5	-	mg/dL	0.1–0.7
AST (GOT)	45	H	U/L	9–37
ALT (GPT)	66	H	U/L	3–49
ALP	244	-	U/L	104–338
γ-GT	44	-	U/L	6–71
Triglycerides	352	H	mg/dL	66–213
HDL cholesterol	41	L	mg/dL	45–55
LDL cholesterol	97	-	mg/dL	70–139
LDL/HDL ratio	2.4	-	-	not established
Sodium	140	-	mmol/L	139–149
Potassium	2.9	L	mmol/L	3.8–4.8
Chloride	99	L	mmol/L	100–114
Blood urea nitrogen	13	-	mg/dL	7–21
Uric acid	6.8	-	mg/dL	4.3–8.6
Creatinine	1.14	-	mg/dL	0.50–1.20
eGFR	53.2	-	mL/min/1.73 m²	≥60
Calcium	8.8	-	mg/dL	8.0–10.7
Inorganic phosphorus	2.7	-	mg/dL	2.5–3.9
ARC	4.13	-	pg/mL	2.49–21.00
PAC	231.3	H	pg/mL	29.9–159.0
PAC/ARC ratio	56	H	-	0.0–39.0
C-reactive protein	0.35	H	mg/dL	0.0–0.20
Glucose	227	H	mg/dL	70–110
HbA1c (NGSP)	7.5	H	%	4.6–6.2

**Figure 2:**
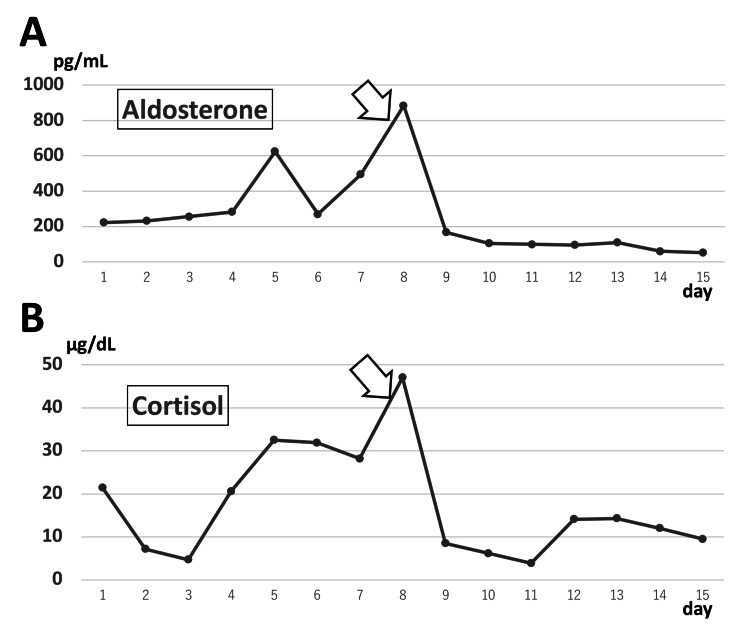
Chronological changes in hormone levels during the clinical course. (A) Plasma aldosterone concentration and (B) serum cortisol levels are shown. Both hormone levels were elevated preoperatively and showed a marked and rapid decline following laparoscopic adrenalectomy performed for therapeutic and diagnostic purposes (indicated by the arrow). Reference ranges were as follows: plasma aldosterone concentration, 29.9–159.0 pg/mL; serum cortisol, 4.30–22.40 µg/dL. The day of admission was defined as day 1.

The chronological changes in aldosterone and cortisol levels are shown in Figure 2. Postoperatively, the patient had an uneventful clinical course and was discharged one week after surgery. The patient has since been managed with appropriate medical hormone replacement therapy. Follow-up evaluations demonstrated stable hormone profiles without evidence of recurrent hormone excess, and the patient remained free of adrenal-related symptoms. No new adrenal lesions or disease progression were observed on follow-up imaging. The patient has remained clinically stable without any complications for 18 months after surgery.

Pathological findings

**Figure 3:**
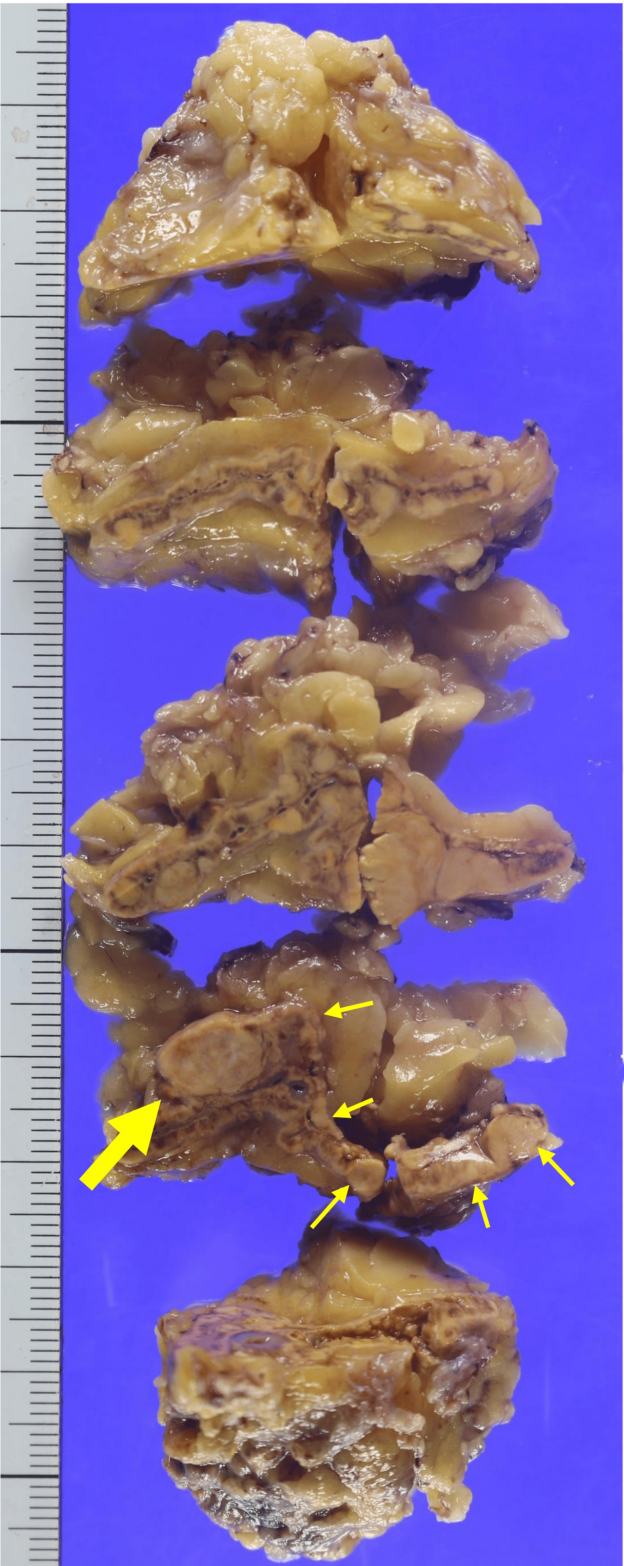
Gross pathological findings of the resected adrenal gland. Gross appearance of the resected adrenal gland after serial sectioning in the frontal plane. A well-circumscribed, spherical, milky-white nodule measuring more than 1.0 cm in diameter is identified (thick yellow arrow). In addition, multiple macroscopic hyperplastic nodules are diffusely distributed throughout the surrounding adrenal cortex (thin yellow arrows).

On gross examination, the resected adrenal gland was serially sectioned in the frontal plane for evaluation. As shown in Figure 3, a well-circumscribed, spherical, milky-white nodule measuring more than 1.0 cm in diameter was identified, corresponding to the area indicated by the thick yellow arrow in the gross sectioning diagram. In addition, multiple macroscopic hyperplastic nodules were diffusely observed throughout the surrounding adrenal cortex, as indicated by thin yellow arrows.

**Figure 4:**
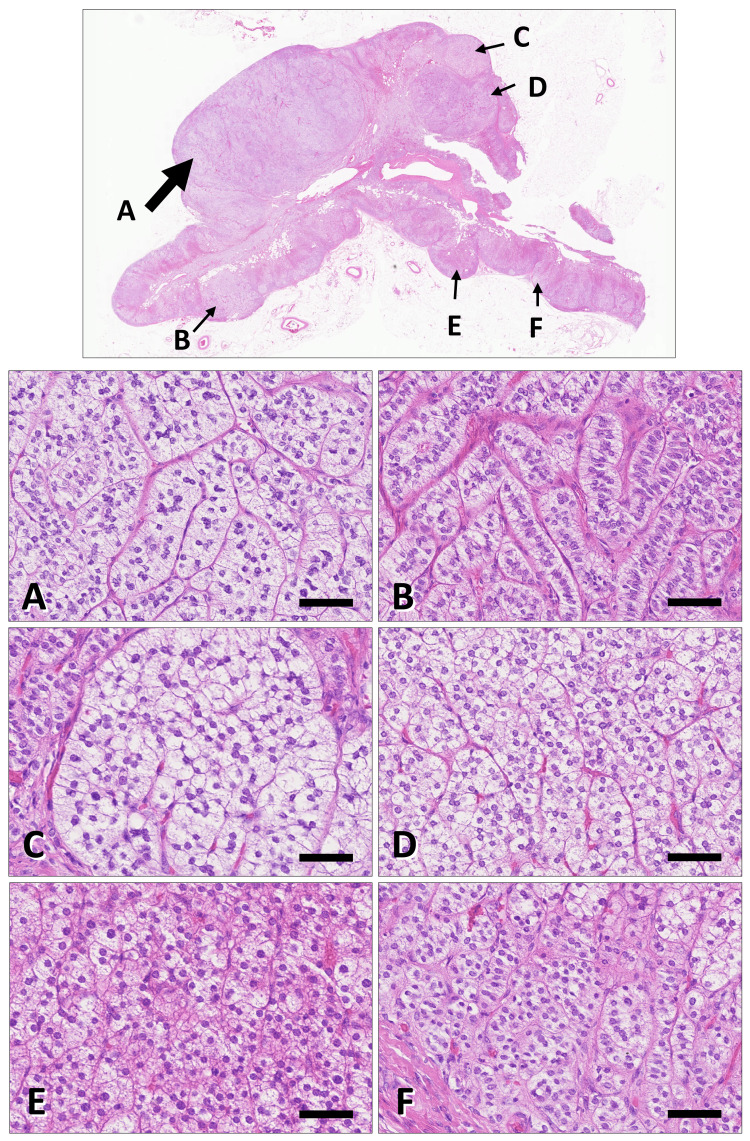
Representative histopathological findings of the resected adrenal gland. Representative hematoxylin and eosin–stained sections of the adrenal gland. Upper panels (low magnification) show the largest nodule (arrow A), which is continuous with the adjacent adrenal cortex and lacks a distinct fibrous capsule, while exhibiting expansile growth with a clear demarcation from the surrounding tissue. Multiple smaller nodules measuring less than 1.0 cm in diameter are present within the background adrenal cortex (arrows B–E). The area indicated by arrow F shows morphologically unremarkable adrenal cortex. Lower panels (higher magnification) demonstrate that nodules A–E are composed of tumor cells with slightly increased cellularity and scattered enlarged or occasionally multinucleated nuclei. The cells show abundant clear to weakly eosinophilic cytoplasm and are arranged in trabecular to solid or nested patterns, with no appreciable morphological differences among nodules. Mitotic figures are rare, and no tumor necrosis or vascular invasion is observed. In contrast, the area indicated by arrow F preserves the normal adrenal cortical architecture, with discernible zona glomerulosa, zona fasciculata, and zona reticularis. Scale bars: 75 μm in panels A–F.

Histologically, multiple adrenal nodules were identified, including one dominant lesion and several smaller nodules within the background cortex (Figure 4). These nodules shared a uniform histological appearance on hematoxylin and eosin staining and were clearly distinguishable from the surrounding non-neoplastic adrenal tissue. In contrast, areas of the adrenal cortex outside the nodules retained normal cortical architecture.

Analysis of ARMC5 gene alterations

**Figure 5:**
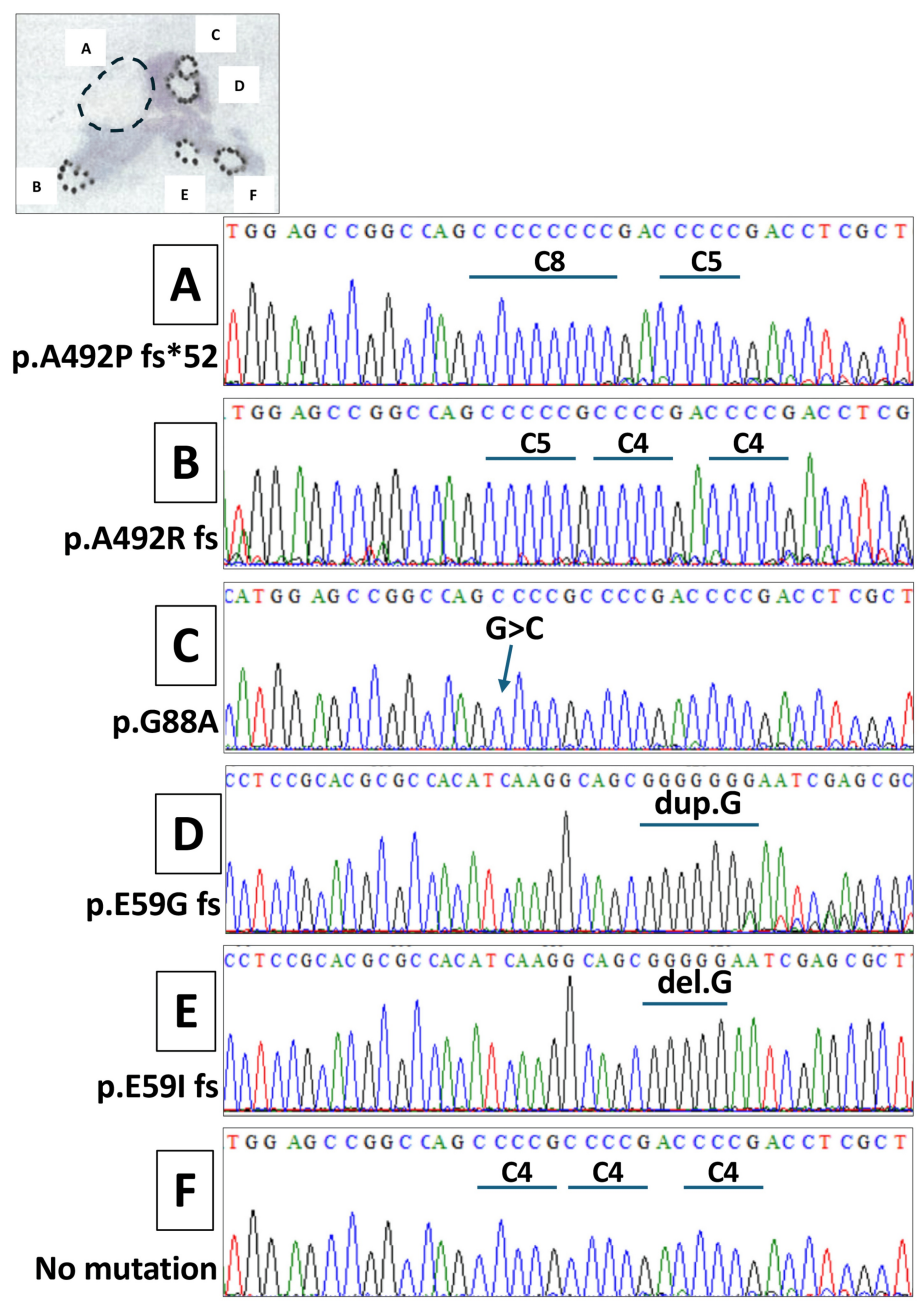
Nodule-by-nodule analysis of ARMC5 gene alterations Summary of ARMC5 gene mutations identified by direct Sanger sequencing of microdissected tissue samples obtained from individual adrenal nodules. Distinct ARMC5 mutations are detected in nodules A–E, whereas no mutation is identified in area F, which shows no histological abnormalities. The largest nodule (nodule A) harbors a p.A492Pfs*52 mutation; nodule B, a p.A492Rfs mutation; nodule C, a missense mutation (p.G88A); and nodules D and E, frameshift mutations p.E59Gfs and p.E59Ifs, respectively.

To investigate the presence of ARMC5 gene alterations on a nodule-by-nodule basis, tissue samples corresponding to areas 1-6 were obtained by microdissection from formalin-fixed, paraffin-embedded sections. Genomic DNA was extracted from each microdissected sample. Primer sets covering exons 1 through 6 of the ARMC5 gene were designed (Table 2), and the amplified PCR products were subjected to direct Sanger sequencing. As a result, distinct ARMC5 gene alterations were identified in each of the nodules 1-5 (Figure 5).

**Table 2:** Primer sets used for PCR amplification and sequencing of ARMC5 exons Primer sets used for PCR amplification and direct sequencing of ARMC5 exons 1–6. All primers were designed to cover the entire coding regions of the respective exons. PCR products were subjected to direct Sanger sequencing.

Exon	Amplicon	Primer sequence (5′–3′)	Annealing temperature (°C)	PCR product size (bp)
Exon 1	Amplicon 1a	F: GATTCTCCCTCGCCTCTTCT	60	491
		R: GAGCTTCTCACGCCTACCTC		
Exon 1	Amplicon 1b	F: GTCGGACTTCTGGGCTGTTC	60	456
		R: CTCAGGGGTGTCTCGTTGGT		
Exon 1	Amplicon 1c	F: TTTCCCTGTCTTCCAGTTCC	56	655
		R: ACGTTATTCGGGATAGGAC		
Exon 2	Amplicon 2	F: AGGGGTTAGACACCTCACAAG	60	204
		R: CACTCAAGCCTTTCTTCTGC		
Exon 3	Amplicon 3a	F: GTAAGAGGCTGTGAGTTGG	58	507
		R: TCATCTACCAGCACCTCCAC		
Exon 3	Amplicon 3b	F: GATCCTGCCAACCACCTGTGT	60	500
		R: CACTGCTCACCTCCACCAAG		
Exon 4	Amplicon 4a	F: TTGGCTCTGGGTTTCAGTCTC	64	629
		R: GAGTGGGAAGGTGAGGTTCT		
Exon 4	Amplicon 4b	F: GGCCTGCTGACCTATGTGAC	60	669
		R: CAGAAGGGCTCCTTGGTCTA		
Exon 5	Amplicon 5	F: GTCTCACTCACCCACCTGT	60	441
		R: ACAGTGGGAGACACAGGTC		
Exon 6	Amplicon 6a	F: ACACCCGCTCTTCCTCTTCT	60	481
		R: CTCTTCCAGCTCCTCCTCCA		
Exon 6	Amplicon 6b	F: CTGCTGGCCGTTTCCTACTG	60	632
		R: GAACAAGACCCTGCTTGGTG		

Specifically, the largest nodule (nodule 1) harbored a p.A492Pfs*52 mutation, while nodule 2 showed a p.A492Rfs mutation. In nodule 3, a missense mutation (p.G88A) was detected, whereas nodules 4 and 5 carried frameshift mutations, p.E59Gfs and p.E59Ifs, respectively. In contrast, no ARMC5 mutations were detected in area 6, which exhibited no morphological abnormalities on histological examination.

## Discussion

In the diagnostic evaluation of nodular lesions of the adrenal cortex, such lesions are broadly classified into two major categories based on whether they are solitary or multiple, and unilateral or bilateral [2,5]. Multiple bilateral nodular lesions are generally associated with endogenous Cushing syndrome [6]. When the nodules exceed 1.0 cm in diameter, the condition is designated as PBMAD, which is characterized by marked heterogeneity at the morphological, functional, and molecular levels, even within a single patient [11,13-15]. Individual macronodules frequently differ in size, histological appearance, and steroidogenic activity, indicating that adrenal enlargement in this disease does not represent a uniform hyperplastic process [14,15]. Although both germline and somatic alterations of ARMC5 have been established as central pathogenic mechanisms underlying PBMAD [12], the clonal relationships among individual nodules within the same adrenal gland remain incompletely understood. In this context, the present case is notable in that genetic analyses were performed separately on multiple nodules obtained from surgically resected adrenal glands. The identification of ARMC5 mutations in each analyzed nodule supports the concept that ARMC5-associated tumor suppressor dysfunction plays a pivotal role in the development of multinodular adrenal disease [9]. At the same time, the presence of multiple nodules with distinct morphological features raises the possibility that individual nodules may arise through independent or partially divergent clonal events on the background of a shared genetic predisposition.

Notably, in the present PCR-based analysis, ARMC5 mutations were detected in a monoallelic manner in each nodule. This finding suggests the possibility that a pre-existing germline alteration affecting the ARMC5 locus may have been present, with additional somatic events contributing to nodule formation, consistent with a two-hit mechanism of tumor suppressor gene inactivation [8,9]. However, the precise allelic status and clonal evolution of individual nodules require further investigation. The detailed nodule-by-nodule analysis, as performed in the present case, provides a more refined view of the molecular landscape of PBMAD and may help clarify how genetic alterations translate into heterogeneous structural and functional phenotypes. Such an approach may also contribute to a better understanding of the variable clinical presentations and differential hormonal activity observed in this disease.

In addition to hypercortisolism, PBMAD has also been reported as a cause of secondary hypertension through its association with primary aldosteronism [16-18]. A previous study analyzing 56 patients with primary aldosteronism demonstrated germline ARMC5 mutations in approximately 40% of cases, and notably, a substantial proportion of mutation-positive patients were reported to have severe obesity [19]. In this context, the present case is clinically concordant with these observations, as primary aldosteronism was initially identified as the cause of hypertension, and the patient exhibited marked obesity. These findings further support the notion that ARMC5-associated adrenal disease may underlie a broader spectrum of endocrine and metabolic phenotypes than previously recognized.

## Conclusions

This study demonstrates that PBMAD is characterized by genetically and functionally heterogeneous adrenal nodules, as evidenced by the presence of distinct ARMC5 mutations in individual nodules within the same adrenal gland. These findings support a model of nodule-specific clonal expansion arising on a permissive background of adrenal hyperplasia, rather than uniform genetic alteration across the cortex. The coexistence of Cushing syndrome and primary aldosteronism in this context further underscores the biological and functional complexity of PBMAD. Collectively, our results emphasize that careful histopathological mapping combined with nodule-resolved molecular analysis is indispensable for elucidating the pathogenesis, intraglandular heterogeneity, and clinical diversity of PBMAD.
